# 

**Published:** 1900-11-15

**Authors:** 


### ANNOUNCEMENTS.

### THE DETROIT DENTAL SOCIETY.

The Detroit Dental Society held the first meeting of the season at the Wayne Hotel, October 22d. No papers were read, but about sixty sat down to a splendidly served banquet, at which Dr. Geo. Field served as toast-master, which is equivalent to saying that there were some good and witty toasts. There were reminiscences by the old men and music by the younger men. Under the management of President Young this society will do some good work this year. A program of the year's work will soon be issued.

### THE WEST VIRGINIA STATE DENTAL ASSOCIATION.

The West Virginia State Dental Association met August 30th and 31st at Wheeling. The following officers were elected for the ensuing year: President, G. M. McNeeley; Vice President, J. M. McVore; Secretary, W. B. McKee; Treasurer, W. McKinley.

### THE SOUTHWESTERN MICHIGAN DENTAL SOCIETY.

The Southwestern Michigan Dental Society met at St. Joseph, September 10th, with the Northern Indiana Dental Society as its guest. The meeting was one of good interest. We shall publish the papers read in the December issue. The following officers were elected: President, N. E. Hooper; Vice President, E. A. Honey; Secretary, C. Johnson; Treasurer, C. E. Burchfield. The next meeting will be held in April at Battle Creek.

